# The impact of linguistic vs. cultural imperialism on language learning

**DOI:** 10.3389/fpsyg.2024.1438849

**Published:** 2025-01-29

**Authors:** Somayeh Razmjoo Moghadam, Ghasem Barani

**Affiliations:** Department of English, Ali Abad Katoul Branch, Islamic Azad University, Ali Abad Katoul, Iran

**Keywords:** cultural imperialism, language acquisition, language learning, linguistic imperialism, review study

## Abstract

The current study systematically reviewed selected literature on background, current conceptualization, and direction of the issues of linguistic and cultural imperialism in publications of applied linguistics and language teaching to determine themes in the field. To do this, based on the inclusion/exclusion criteria, provided in the PRISMA Chart, 30 most updated and recent articles (mainly since 2020) were selected from the 5 main publications in the field through the advanced search engines. Then, two raters used coding books to screen and code necessary quantitative and qualitative data based on which, a total of 989 general coding schemes and categories were elicited from the coding of the main themes, trends, and findings of linguistic and cultural imperialism. Overall, the main themes of the study were provided in the form of the concepts and perspectives of linguistic and cultural imperialism, informed by the historical directions and the influence of the colonial era. Moreover, the role of power relations and prevailing linguistic dominance in supporting dominant languages and the influence of linguistic and cultural imperialism on L1 acquisition were presented and discussed. Since language imperialism can impact L1 language acquisition by marginalizing local languages and threatening them, each community needs to follow its practical language policy and plans to revitalize and support its languages and cultures. It was suggested that the intersection of linguistic and cultural imperialism impacts social and language identity which can lead to neo-imperialism, colonization, and language hierarchization. The study puts forward some recommendations and suggests future directions to reinforce language rights through different parties with the integration of a human rights perspective in language preservation efforts as the main actions that can be done to improve language awareness of the people. Policy-makers and language decision-makers can follow these guidelines to preserve the legal aspects of the language and cultural identity and utilize foreign languages in more rational and non-threatening ways.

## Introduction

Language learning is not a neutral process per se, since through learning the language, some linguistic and cultural concepts are transmitted as the by-products of the language learning process. The intersection of linguistic and cultural imperialism through political and economic hegemony is evident in the dominance of English as a tool for imposing Western ideologies and culture on non-English-speaking populations (Phillipson, 2008). Many languages in the world are impacted by issues like linguistic and cultural imperialism which can change the way they are taught, regarded in the world, and even transferred to the next generation. Linguistic imperialism is the way some dominance can take place among languages due to power relations which can often result in discrimination and inequality (Phillipson, 2012). Due to linguistic and cultural imperialism, some languages become minority and even endangered languages which can lead to their disappearance.

Though linguistic imperialism has been debated for over two decades in language policy fields, it has always shown its impact on language acquisition and learning and how it is prioritized (Yamada, 2023). For example, the way the English language has gained prominence and become the dominant language of the world has been investigated from different perspectives on language planning or merely incidental to colonialism and globalization (Spolsky, 2019). Edwards and Edwards (2011) also discussed the way linguistic imperialism impacted L1 language acquisition in light of issues like the preference for English over Gaelic and challenges faced by non-native English speakers. Another prominent case of linguistic and cultural imperialism comes from McCain and McCain (2018) who argued that local officials in the Napoleonic state acted as cultural intermediaries, advocating for linguistic particularism amidst imperial pressures and they promoted standard French over regional languages, associating the latter with lower status. However, Phillipson (2012) seems to be more concerned with the nature of language imperialism and argues it is not a mere conspiracy, but an entity underpinned by explicit or implicit language policies that intentionally advantage certain languages at the expense of others.

Linguistic imperialism needs careful attention and immediate action from the part of the different parties to preserve language and culture as Astanina and Kuznetsov (2020) stated that the dominance of more powerful languages (particularly English) can impact L1 language acquisition by potentially overshadowing native cultural values and heritage and resulting in different kinds of language shift and death. One prominent example of linguistic imperialism comes from Rose and Conama (2018) in the treatment of Irish Sign Language (ISL) where users face discrimination and inequality due to policies and practices (e.g., linguicism, audism, and the denial of linguistic rights) that fit the linguistic imperialism paradigm. Linguistic imperialism paradigm includes several components as structural inequality, historical context, linguicism, cultural and educational impact, globalization effects resistance and critique (Phillipson, 1992). Therefore, ISL’s rules and regulations are overshadowed by more dominant languages (in this case English) which control its policies, rules, and resources.

Linguistic imperialism is not limited to specific contextual, cultural, linguistic, and historical contexts. Within the contemporary world, many cases of linguistic and cultural imperialism show that major languages negatively impact minority languages (Pujasari and Hikmatullah, 2023). There is much evidence of linguistic and cultural imperialism even in some countries which give enough support and care to their national languages and may seem not to be directly affected by the dominance of more dominant languages (Ahmed et al., 2023; Guo, 2020; Montrul, 2022; White et al., 2023). These studies show that linguistic and cultural imperialism can impact linguistic shift and identity formation within a nation which can have negative consequences on the future of the nation, including the national and local languages. El-Haddad (2022) believes that acknowledging diverse languages and cultures fosters unity and resilience against crises. Such socio-cultural and linguistic stance is believed to affect EFL learners’ language proficiency (Amir Abadi and Razmjoo, 2022). Therefore, more plans and policies are needed to protect the languages of the world and their sociocultural resources.

The way national and local languages can jeopardize minor languages and endanger them can also be highlighted. Many nationalistic movements within a country emphasize linguistic homogeneity within nation-states which can eventually disadvantage speakers of minority languages (Van Dongera et al., 2017). For example, the rise of nationalism across Europe and elsewhere has led to local or national linguistic imperialism, where political leaders prioritize the dominant ethnic or linguistic group’s language, often at the expense of minority languages.

Due to these issues, linguistic and cultural imperialism remain as valid constructs, affecting minority languages through discriminatory policies and practices. Cultural imperialism is associated with different negative outcomes which can eventually lead to the identity loss of the speakers of a language and its ultimate shift (Du Bois et al., 2024) Therefore, researchers continue to explore their impact, emphasizing the need for equitable language policies that recognize and support linguistic diversity. The current study performs a review study to explore the background, current conceptualization, and direction of the issues of linguistic and cultural imperialism and their impact on language acquisition and their future themes, alongside the gaps, findings, and the following trends in the field which are going to determine the themes in the field to provide some suggestions and recommendations in the field. Therefore, more studies are needed in the field since the cohesive reviews of this particular nature are currently lacking which can recapitulate the main trends of linguistic and cultural imperialism and their impact on language acquisition in the literature as well as the main findings on the impact of linguistic and cultural imperialism on language acquisition.

Drawing upon the cited gap in our knowledge of idea of linguistic and cultural imperialism and their impact on language acquisition, as a review study, the current study utilizes the information coming from multiple sources and different phases of the study to shed light on the main themes to address the gaps to present the findings and the following trends in the field which are going to determine the themes in the field and finally present some suggestions and recommendations in the field to improve the global understanding about the issues of linguistic and cultural imperialism and their impact on language acquisition. Therefore, the study answers the following main research question:

RQ1: What are the main trends and findings of the impact of the linguistic and cultural imperialism on language acquisition in the literature?

## Methodology

The current study systematically reviewed the related literature on background, current conceptualization, and direction of the issues of linguistic and cultural imperialism to come up with the main themes to address the gaps review the findings, and shed light on the future trends in the field which are going to determine the themes in the field and provide some suggestions and recommendations in the field. Following the queries on main journals of the field, and those that publish language imperialism articles, and strategies for inclusion and exclusion, the final 30 articles were selected from among the most important and relevant findings, and the common themes of these studies were explored and presented in the study.

### Search strategy

Based on keywording and mapping, the search was done on some main publications of applied linguistics and language teaching (e.g., WoS, Springer, Elsevier, Sage Publication, Taylor and Francis, and other related journals that publish language imperialism articles). To do this, 30 articles were selected from among 75 papers in these databases through the advanced search engines of these main publications in the field of applied linguistics and language teaching using the following Keywording and mapping strategies of the study, based on utilizing AND option, in order to ensure the comprehensiveness of the search findings on linguistic or cultural imperialism and through the options provided in their search engines:

1. Linguistic Imperialism
2. Cultural Imperialism
3. Theoretical Examination
4. Language Acquisition
5. Language Learning

### Inclusion/exclusion criteria

Due to the large number of studies in the field, the reviewed studies had some basic criteria and considerations to be included in the study as follows:

1. limiting the literature to only studies in the English language
2. selecting the most updated and recent literature
3. studying the linguistic imperialism and cultural imperialism of dominant and imperialist languages (e.g., English) and their impact on minor languages
4. selecting from among the main journals and book chapters in the field and related journals that publish language imperialism articles based on some major considerations like the quality of the journal, the novelty and comprehensive design of the article, their investigation of the language and cultural imperialism as the main themes of the study, and also how they added to the findings of the study
5. not excluding articles based on their methodologies (i.e., both quantitative and qualitative studies were included in the study and only a types of article, namely meta-analysis, was excluded)

Based upon these criteria, 30 articles were randomly selected from among those papers published in the main journals and book chapters in the field that investigated language and cultural imperialism and how they impacted the language. According to Zou et al. (2020), limiting journals to high-quality and trusted ones will lead to more robust and reliable results. These articles were mainly published since 2020, as some studies covered the trends and developments in the field before that time (e.g., Golonka et al., 2014; Macaro et al., 2012; Philips, 1987; Kalyaniwala and Ciekanski, 2021). However, in cases where there were influential studies that had a great impact on the field, they were included in the study (e.g., Phillipson’s work and some main book chapters). The articles were selected based on the quality of the journals, the novelty and comprehensive design of the article, their investigation of the language and cultural imperialism as the main themes of the study, and also how they added to the findings of the study.

**Figure 1:**
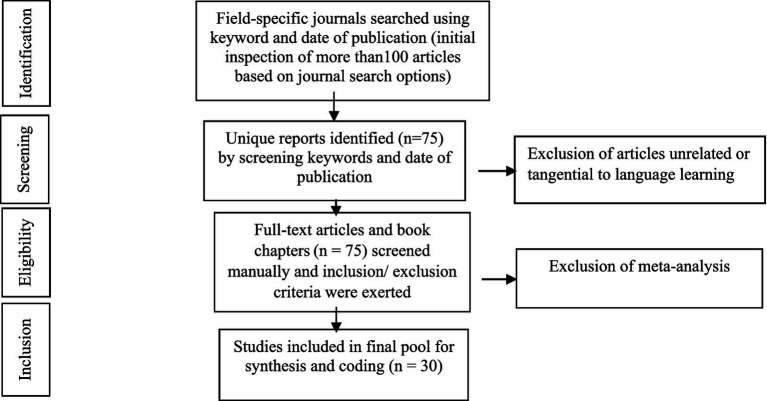
The PRISMA Chart of the processes for the creation of the report pool (originally adopted from Moher et al., 2009).

These criteria were applied to the articles based on the scrutinization and analysis of 75 articles by two researchers and the final 30 articles were included to be reviewed in the study. The PRISMA chart (Figure 1. originally adopted from Moher et al., 2009) represents processes for the creation of the report pool in this study.

### Coding

The final 30 articles selected for the review were coded by two coders through a categorization scheme that was developed based on the keywording (as the process of assigning keywords or key phrases to data to make it ready to analyze) and mapping strategies (the process of creating a structured representation of keywords related to a content area to organize keywords thematically), previously explained in the search strategy and Prisma Chart (Figure 1). Then, the raters used coding books to screen and code necessary quantitative and qualitative data based on which, a total of 989 general coding schemes and categories were elicited from the coding of the main themes and conceptualizations of linguistic and cultural imperialism and their different trends and findings and the major themes which represented the issue from different perspectives (See Table 1). The coding book listed all the codes used in the analysis along with their definitions and examples and served to maintain consistency among researchers to apply coding methods effectively and transparently and provided clarity on how to apply each code to systematically identify and interpret patterns in their data, enhancing the reliability of their findings. The codebook helps researchers 37. Then, to ensure consistency in the coding and interpretation of the data, inter-coder reliability coefficients were performed which showed an acceptable reliability coefficient of 84.1 for the emergent themes. This is well beyond 0.70 as the conventional threshold level (Bos, 1989). Based on the coding of the two coders, the following emergent themes and conceptualizations of linguistic and cultural imperialism were formulated (Table 1). According to the table, correlating themes refer to themes that exhibit a relationship or connection with one another which were kept by the researchers. On the other hand, non-correlating themes are those that do not show a significant relationship or connection with each other. These non-correlating themes were omitted from among the available data.

**Table 1:** The main themes and conceptualizations of linguistic and cultural imperialism.

General coding scheme	Total categories	Correlating	Non-correlating	Inter-coder reliability
Concepts and perspectives of linguistic and cultural imperialism	266	217	49	81.5
The influence of the colonial era on L1 acquisition	202	170	32	84.1
The Role of power in determining Dominant Languages	147	122	19	82.9
Prevailing linguistic dominance	93	75	18	80.6
The intersection of linguistic and cultural imperialism impacts social and language identity	58	44	14	75.8
Neo-imperialism and language policy	35	27	8	77.1

## Results

Here, major themes regarding the current conceptualization and issues of linguistic and cultural imperialism and their impact on L1 acquisition are going to be presented and discussed through analysis of selected key papers. These emergent themes were presented in Table 1. as a general coding scheme and they will be elaborated in the subsequent parts.

### Concepts and perspectives of linguistic and cultural imperialism

By definition, linguistic imperialism refers to the dominance of one language over others, often driven by economic, political, and cultural power dynamics (Rose and Conama, 2018). Linguistic imperialism can have severe consequences on languages and it can lead to language shift through the erosion of indigenous languages. When a dominant language (often associated with power and prestige) infiltrates a community, speakers gradually abandon their native language in favor of the dominant one. Moreover, it can end in the loss of linguistic diversity. As dominant languages spread, minority languages face endangerment or extinction. This loss affects cultural heritage and identity. There should be some guiding rules for maintaining languages and efforts to maintain and revitalize endangered languages become crucial to counter linguistic imperialism (Rose and Conama, 2018).

Cultural Imperialism is closely related to linguistic imperialism and it involves the imposition of one culture’s norms, values, and practices on another culture and it often accompanies linguistic imperialism (Du Bois et al., 2024). Du Bois et al. (2024) stated that cultural imperialism disrupts traditional practices, beliefs, and customs. Individuals may experience a sense of disconnection from their cultural roots which is known as identity fragmentation. Exposure to dominant cultures leads to hybrid identities. People navigate between their indigenous culture and the influences of the dominant culture. Cultural imperialism marginalizes minority cultures, leading to feelings of exclusion and powerlessness. Moreover, as Du Bois et al. (2024) declared, dominant cultural norms may shape educational systems, affecting how history, literature, and art are taught which can perpetuate identity crises.

The literature on language and cultural identity includes several striking studies which not only used methodologically and theoretically robust and sound procedures but also presented full discussion and informative conclusions about the topic. Among these studies is the case study of Nigerian languages and identity crises by Udoh and Emmanuel (2020) in Nigeria with over 500 languages facing linguistic and identity challenges. The researcher concluded that in Nigeria, English as the official language coexists with major indigenous languages (e.g., Hausa, Yoruba, Igbo) and numerous minor languages. In this setting, linguistic, political, ethnic, and educational identity crises arise due to this linguistic diversity.

Another study contributing to the field is the study on the Malakar identity shift in Kerala (Mannathukkaren, 2023). In this setting, language and cultural shifts endanger communities. It was concluded that minor changes in practices and verbal repertoires can lead to a complete transformation of community identity. As the researcher found that bilingual or multilingual speech communities experience language shifts, he put forward some general guidelines. These guidelines focus on understanding the complexities of identity, consumption, and social differentiation within the context of Kerala’s socio-economic landscape to reduce the negative effects the language and cultural imperialism.

#### Historical directions of linguistic and cultural imperialism

Linguistic and cultural imperialism have historically been prevalent phenomena, with English often playing a dominant role. It was through the groundbreaking works of Phillipson (1992, 2009, 2012, 2014) that linguistic imperialism flourished as an independent and multidimensional field of study. Phillipson’s work highlights the ongoing issue of linguistic imperialism, where English’s global dominance perpetuates Western ideologies. This dominance can lead to negative attitudes towards native languages, as seen in the case of Assamese speakers feeling inferior due to English’s influence (Ahmed et al., 2023).

Different studies have shown that due to the dominant nature of linguistic and cultural imperialism, can significantly impact L1 acquisition in various ways (Phillipson and Kabel, 2024). It can be done through language hierarchizing and changing social values. Aydinli and Aydinli (2024) believed that linguistic imperialism refers to the dominance of one language over others. For instance, English and French have historically been propagated as dominant languages in colonized countries and more recently in many European nations. Moreover, the social value of a language can influence its learning. Generally, languages associated with power, academia, or international communication are often prioritized. Conversely, minority languages may be marginalized, affecting L1 acquisition.

Recently, various theoretical approaches, such as neo-imperialist, colonial, and postcolonial discourse and cultural imperialism shed light on the complexities of modern imperialism, including linguistic imperialism (Yazovskaya and Gudova, 2020). The spread and retention of English’s power in communication, business, academia, and education further reinforced its imperialistic nature (Lai, 2021). These dynamics underscore the need to preserve local languages alongside English to counter the impacts of linguistic neo-imperialism. These studies can focus on more recent trends and ideas like language shift, death, and hierarchizing languages in a way that such concepts would lead to the development of knowledge, competence, and skills among the language users to try to protect local languages as treasures which can add to the current global body of knowledge. To minimize the impact of linguistic and cultural imperialism on minority languages, Ayixiren and Zhao (2024) stated that there should be more language policies and integration to support the dominant languages. For example, national language policies play a crucial role in supporting the languages since some policies prioritize dominant languages and they may hinder L1 maintenance. Many policies including integration models should recognize multilingualism and promote L1 alongside L2 learning (Rose and Conama, 2018).

### The role of power in determining dominant languages

Different studies have shown that linguistic and cultural imperialism and the power of more dominant languages can significantly impact L1 language acquisition (Ahmed et al., 2023; Fauzan, 2023; Saba and Siddiqui, 2023; Schlechtweg et al., 2023; Zeng et al., 2023). The dominance of English as an imperialist language in various global domains can lead to the prioritization of English over local languages in education (Saba and Siddiqui, 2023). Saba and Siddiqui (2023) stated that this phenomenon, known as “English fever,” reflects a strong desire to learn English, potentially overshadowing the importance of acquiring one’s native language. The perpetuation of English as a global lingua franca can create social inequalities between English speakers and non-speakers (Schlechtweg et al., 2023). Additionally, the global dominance of English can influence societal norms, values, and beliefs, particularly among younger generations exposed intensively to English (Zeng et al., 2023). Understanding linguistic imperialism and its effects on cultural identity is crucial in evaluating its impact on L1 language acquisition.

#### Evidences of language dominance on different languages

There are many different cases of major and more dominant language dominance and imperialism which can harm other languages. These studies have shown that the dominance of languages like English can lead to a negative attitude towards native languages, such as Assamese in India, potentially endangering them in the future (Ahmed et al., 2023). Linguistic and cultural imperialism can have a severe negative influence on native languages. Exposure to an L2 can negatively impact specific areas of L1 grammar. For instance, Kelly (2023) indicated that L1 influence can be negative due to differences in linguistic properties between languages, and not all languages share the same structures, making L2 learning challenging. However, other mediating factors (e.g., gender and providing feedback) are also known to be important in the ultimate grammatical performance of the learners (Izadpanah et al., 2023; Shokri, 2022). White et al. (2023) also asserted that such an impact can be seen on L1 Afrikaans children’s acquisition of negation. Furthermore, the spread and retention of English as a global lingua franca can perpetuate linguistic neo-imperialism, reinforcing its dominant position in various domains, including education. Overall, these influences highlight the complex interplay between linguistic imperialism and L1 language acquisition.

#### Consequences of language imperialism

Regarding its consequences as studied by different researchers (Kamusella and Ndhlovu, 2018; Lai, 2021; Nitisakunwut et al., 2023; Soto-Molina and Méndez, 2020; Yamada, 2023), linguistic imperialism raises concerns about the impact on L1 language acquisition due to the dominance of English. Soto-Molina and Méndez (2020) contended that the spread of English as an imperialist language can compromise the cultural integrity of non-native speakers. In Indonesia, the overwhelming desire to learn English has led to policies promoting English-only instruction in schools, potentially affecting the acquisition of the first language (Nitisakunwut et al., 2023). Scholars advocate for cultural awareness and intercultural competence to counter linguistic imperialism and emphasize the importance of recognizing cultural diversity in English language teaching (Kamusella and Ndhlovu, 2018; Lai, 2021; Nitisakunwut et al., 2023; Soto-Molina and Méndez, 2020; Yamada, 2023). The global dominance of English can lead to social inequalities and reshape cultural identities, highlighting the need for efforts to address these issues (Kamusella and Ndhlovu, 2018; Yamada, 2023). Ztf (2017) also asserted that language imperialism can impact L1 language acquisition by marginalizing local languages. Cultural imperialism through language, as it was evidenced in this study, also influenced how individuals acquire their first language.

### The intersection of linguistic and cultural imperialism

As Phillipson (2008) truly pointed out, the intersection of linguistic and cultural imperialism is evident in the dominance of English as a tool for imposing Western ideologies and culture on non-English speaking populations. He further pointed out that English as the official language of over 60 countries, plays a crucial role in establishing political and economic hegemony, reminiscent of political imperialism. The complex relationship between standardized languages and regional dialects highlights the tension between cultural particularism and conformity to dominant linguistic norms.

#### Linguistic imperialism through educational reinforcement

This linguistic imperialism is further reinforced through educational materials, like textbooks, which promote the socio-cultural ideologies of English-speaking nations while alienating local cultures which is prevalent within the literature (Nitisakunwut et al., 2023; Wang, 2017). These studies mainly review the challenges of socio-cultural and vernacular influences on L1 learners acquiring English (L2) in different contexts, suggesting practical solutions for language learning issues.

In the Iranian context, Arab and Rastgou (2022) explored the textbook Prospect 3 to detect different kinds of language and cultural inconsistencies based on a critical analysis by Nation and Macalister’s Four-Strand Framework.

Linguistic and cultural imperialism have been investigated through different lenses as the study of migration and diaspora, policies on bilingualism and multilingualism, technological impact, historical lens analysis, and issues like policy effects, cultural identity, language subordination, coordination, language shift, and even language death.

Such studies can be done through different themes and methodologies like case studies analysis, theoretical frameworks, language proficiency studies, ethnographies, or even document analysis and deep interviews with informed people through phenomenology and grounded theories.

### Impacts on social, economic, and language identity

The impact of language and cultural dominance on language identity can be evidenced in the disappearance of native languages in different parts of the world. For example, such power relations leading to the subordination of languages and cultures can be seen in former British colonies’ education systems (Zeng and Yang, 2024).

Social Identity Theory (SIT) is an idea closely related to the notion of language imperialism and identity maintenance. Proposed by Tajfel and Turner (2004), SIT explains how group membership influences individual identity. Recognizing diverse identities is essential for unity. Moreover, implementing dual-language programs at the basic education level can promote linguistic and cultural diversity which can lead to more informed language learners through improved understanding of social, cultural, and language identity. These programs should empower all language communities.

Linguistic Shifts and Identity crises are among the major consequences of language imperialism among different nations. The relationship between language loss and identity crises amongst indigenous communities is generally studied by different researchers to become a trend in the field (Huang and Chan, 2024). among these studies, some major studies have addressed the ideas of language endangerment and death and Factors contributing to the extinction of indigenous languages.

These studies can address some societal implications like civic engagement impacts on improving the level and quality of the languages, economic status which can be seen as a way to improve the position of the language and culture, and social cohesion effects to preserve the languages and cultures of a country.

### Prevailing linguistic dominance

Different studies have addressed the role of dominant languages and cultures in the manifestation of different ideas like colonization, neo-imperialism, and language subordination processes throughout the world. Among the most important studies in the field of language and cultural dominance is the case study of Nigerian languages and identity crises by Onanuga (2024) in Nigeria with over 500 languages facing linguistic and identity challenges. The researcher concluded that in Nigeria, English as the official language coexists with major indigenous languages (e.g., Hausa, Yoruba, Igbo) and numerous minor languages. In this setting, linguistic, political, ethnic, and educational identity crises arise due to this linguistic diversity. Moreover, the study in Malakar Identity Shift in Kerala showed that language and cultural shifts endanger communities. It was concluded that minor changes in practices and verbal repertoires can lead to a complete transformation of community identity. As the researcher found that bilingual or multilingual speech communities experience language shifts, he put forward some general guidelines to reduce the negative effects the language and cultural imperialism.

Studies on the way major dominant languages of the world have impacted minor languages can be acknowledged to address such power relations leading to language subordination and dominance. Among these studies, we can refer to the impact of English colonization on Native American languages (Almas and Mazhar, 2024), the influence of French language in African countries (Ball et al., 2024), the impact of English language policy in international schools on L1 (Liu et al., 2024), and the influential study on how American pop-culture influences English language learning worldwide (Foner, 2024) tried to address such unequal and dominating language relationships.

The dominance of English over indigenous languages and the problems raised in acquiring the native languages of the people is another main issue in the field, addressed by different researchers (Mathew, 2022; Saba and Siddiqui, 2023; Pujasari and Hikmatullah, 2023; El-Haddad, 2022). This dominance can lead to the marginalization of indigenous languages and cultures, affecting the acquisition and preservation of L1 languages (Pujasari and Hikmatullah, 2023). This phenomenon raises concerns about compromising cultural integrity and perpetuating Anglo-American hegemony (Mathew, 2022). The perceptions of speakers of English as a Second and Foreign Language regarding linguistic imperialism highlight the need for changes to prevent discrimination and uphold linguistic human rights (Pujasari and Hikmatullah, 2023). For instance, in the context of Indonesia, the removal of English language programs from elementary school curricula due to fears of hindering L1 development sparks debates among scholars and educators, emphasizing the importance of English learning for empowerment in a globalized world (Pujasari and Hikmatullah, 2023). Moreover, in post-market reform India, the desire for English schooling as a result of social mobility shifts highlights how linguistic imperialism reshapes language learning patterns (El-Haddad, 2022). Overall, linguistic imperialism can impact L1 language acquisition and usage by influencing language policies, societal and cultural perceptions, and educational practices in diverse cultural contexts (Lai, 2021).

### Studies addressing English dominance on local and national languages and cultures

The use of English alongside local languages can lead to cultural imperialism, as seen in Hyderabad, India (Singh and Gaur, 2020), Indonesia (Sugiharto, 2024), Hong Kong (Lai, 2021), Iran (Khodadady and Shayesteh, 2016), Turkey (Işık, 2008), and many other countries. For example, some case studies in Indonesia (Ztf, 2017; Fauzan, 2023; Sugiharto, 2024) found that the arbitrary combination of national and local languages (e.g., Bahasa) and English led to a form of language imperialism that perpetuates cultural dominance.

### Neo-imperialism, neo-colonialism, and language policy

Neo-imperialism in language policy is evident through the spread and dominance of English globally, impacting various domains like communication, business, academia, and education (Zeng et al., 2023). While some view English as a tool for empowerment and globalization, others criticize its imposition on local languages and cultures (Lai, 2021). As Sah and Fang (2024) declared, the adoption of English as an official language in former colonies has reinforced the political and economic hegemony of English-speaking nations over non-English-speaking populations. Additionally, neo-nationalist ideologies in countries like the United States advocate for monolingual English policies, reflecting a hyper-focus on internal homogeneity and border maintenance (Sah and Fang, 2024). These dynamics highlight the complex interplay between language, power, and imperialism in shaping contemporary language policies and practices globally.

Zeng et al. (2023) studied globalization effect intertwined with English linguistic neo-imperialism. It was suggested that English linguistic neo-imperialism can impact language policies in periphery countries, influencing education, communication, and business. Moreover, it was found to reinforce English dominance and also potentially marginalize local languages. However, as the researcher pointed out, some further studies were needed to support or challenge the findings. The study ended with some implications for preserving local languages through providing enough support for them.

#### Educational plans and policies against imperialistic views on language and culture

Pujasari and Hikmatullah (2023) investigated new paradigms on language imperialism and highlighted the role of student-teachers’ voices on English language learning. The study drew upon Phillipson’s ideas on English spread through colonialism and neo-colonialism. She believed that English student-teachers in Indonesia view English teaching as empowerment, not imperialism. She also asserted that these student-teachers acknowledged the activities to promote local cultures and languages globally, challenging the notion of language imperialism. She talked in favor of arguments for promoting English which include intrinsic, extrinsic, and functional aspects. The study provided an alternative perspective on English language and the globalized role of imperialism.

The next study by Lai (2021) speculated the role of English linguistic neo-imperialism in Hong Kong. He also acknowledged Phillipson’s (2012) warning about English linguistic neo-imperialism which could severely impact minority languages. The study provided insightful findings about beneficial ways of English language teaching in global settings whereby local and minority languages and cultures can be respected and preserved, meanwhile, the role of more powerful languages in bringing about positive international changes and collecting the common treasures of the different nations of the world can also be acknowledged. However, the study warned about English linguistic neo-imperialism and how to use English in more justifiable and non-threatening ways.

The next study by Singh and Gaur (2020) elaborated on English linguistic imperialism in neo-imperialism and politics of language and countering colonialism which can reinforce political and economic dominance, impacting language policies in former colonies like Amitav Ghosh’s The Shadow Lines. It was suggested that linguistic imperialism and political imperialism are analogous in that English linguistic imperialism can establish political and economic hegemony. These findings are also supported by the critical discourse analysis of public discourse of Meadows’ study of neo-nationalism in the United States. The study advocated monolingual English use in US and provided some insightful findings about the language policy and how it can result in more equal and just consequences towards less threatening and non-hegemonic and non-imperialist ideas.

## Discussion

Based on the findings of the study and analysis of the most relevant literature, several themes related to the language and cultural imperialism regarding the L1 acquisition were postulated. A main theme was related to the influence of colonial and neo-imperialist perspectives on L1 acquisition. Based on the review of the most important studies, the influence of the colonial era on L1 acquisition is significant, especially in developing countries where the colonial language is favored for education (Zeng et al., 2023). Children in these regions may struggle with literacy if they are not proficient in the language used for instruction, leading to difficulties in learning and interacting with educational content (Yook, 2012). Additionally, the role of L1 in second language learning, or language transfer, remains crucial in a globalized world where multilingualism is common (Hussain, 2023). Studies have shown that L1 can impact the learning order and accuracy of L2 morphemes, challenging the notion of a universal order of acquisition (Murakami and Alexopoulou, 2016). However, provided that language learning follows plans and strategies to minimize the effect of language imperialism, it can have some benefits to improve cross-cultural and multi-lingual identity and understanding of the language learners. Therefore, each community needs to follow its language policy in order to save the minority languages and also improve the language awareness of the nation to keep them both competent, culturally-aware, meanwhile independent nation.

Regarding the role of power in determining dominant languages, different researchers studied its consequences on language learners (Kamusella and Ndhlovu, 2018; Lai, 2021; Nitisakunwut et al., 2023; Soto-Molina and Méndez, 2020; Yamada, 2023) and it was found that linguistic imperialism raises concerns about the impact on L1 language acquisition due to the dominance of English. It was contended that the spread of English as an imperialist language can compromise the cultural integrity of non-native speakers (Soto-Molina and Méndez, 2020). Nitisakunwut et al. (2023) stated that the overwhelming desire to learn English can lead to policies promoting English-only instruction in schools, potentially affecting the acquisition of the first language. Therefore, it was concluded that cultural awareness and intercultural competence are necessary for language learners to counter linguistic imperialism and emphasize the importance of recognizing cultural diversity in English language teaching (Kamusella and Ndhlovu, 2018; Lai, 2021; Nitisakunwut et al., 2023; Soto-Molina and Méndez, 2020; Yamada, 2023).

Another major theme was attributed to the influence of linguistic and cultural imperialism on L1 language acquisition. Yook (2012) studied ESL learners’ learning of English ditransitive constructions with a focus on semantically comparable English and Chinese structures and constructions. Hussain (2023) studied FL influence on second language learning. It was suggested that children find second language learning easier compared to adults and L1 helps in improving language learning and skill development through facilitating communication and peer revision among ESL learners. The next study by Murakami and Alexopoulou (2016) on L1 influence on the acquisition order of English grammatical morphemes, using contrastive analysis hypothesis and error analysis approaches, Thao (2020) studied the role of L1 in L2 learning. Mizza (2014) also provided a Model of Literacy Instruction through comparing L1 and L2. Alonso (2016) focused on recent perspectives like linguistic relativity and cognitive linguistics. Oviedo (2017) in another review study of the approaches and theories in L1/L2 acquisition research, investigated the theoretical Paradigms on L1 Acquisition. Diaubalick et al. (2023) explored L2 transfer of imperfective meaning from one Romance language to another and it studied the influence of L1/L2 linguistic knowledge.

Overall, it can be stated that due to the prevailing globalized linguistic and cultural dominance accompanied with English language and culture, social, economic, and cultural inequalities can reshape cultural identities and opportunities for the access to the knowledge of the world and maintaining the independence of the country, which highlights the need for more studies to address these issues (Kamusella and Ndhlovu, 2018; Yamada, 2023). Furthermore, language imperialism can impact L1 language acquisition by marginalizing local languages and threatening them through language shift and even death (Ztf, 2017). However, in order to keep the minority languages alive and also improve the language awareness of the nation to keep them culturally-aware and independent nation, each community needs to follow its language policy and plans to revitalize the minority languages and support them as the gates to the treasure of language, culture, and livelihood of the nation. Hence, language boundaries and the differences among the languages should be regarded as socially constructed rather than natural and traditional constructs and harmful terminology in linguistic theory in favor of language hierarchization, native-speakerism, and monolingualism which may lead to supremacy and domination of specific languages and groups should be avoided (Cheng et al., 2021; Namboodiripad and Henner, 2022).

It is important to note that the global dominance of English can not only lead to social inequalities and reshape cultural identities through marginalizing local languages and threatening them, but it can also lead to educational neo-imperialism and colonialism in the way that local and national languages are threatened. Therefore, alongside teaching English language and culture, language learners should gain multi-dimensional literacy and be more alert regarding the different ways that language learning can lead to social, political, and cultural domination of more dominant languages, and how to keep such impact at minimum. This can happen by crediting the value of the local and national languages and specifying educational plans and strategies to allocate their protection within the educational objectives. However, more studies on language subordination and hierarchization fields are needed to show how the dominant languages have impacted them socio-politically and globally and threaten their linguistic and cultural treasures.

## Conclusion

Imperialistic and hegemonical sides of English Language teaching in presenting a multi-dimensional dominative and superior language to the public has highlighted the role of power relations and prevailing linguistic dominance in supporting dominant languages and how they influence L1 acquisition. Undoubtedly, linguistic and cultural imperialism impact L1 acquisition by shaping language hierarchies and marginalizing local languages, affecting language policies, and influencing individual identity. Efforts to preserve and value diverse languages are essential for a more inclusive linguistic landscape. It was suggested that the intersection of linguistic and cultural imperialism impacts on social and language identity which can lead to neo-imperialism, colonization, and language hierarchization. Each community needs to follow its practical language policy and plans to revitalize and support its languages and cultures. Different strategies and solutions in the form of policy reforms can be determined by policy-makers in order to minimize the negative effects of linguistic and cultural imperialism on language learners. It can include prioritizing the importance of language preservation policies in linguistically diverse nations. Moreover, through raising awareness of the language members of the society, they can be more aware about their roles in preserving their heritage and mother languages. This can be done through emphasizing the role of media and education in promoting linguistic diversity, especially within the young generations who may be vulnerable to lose their language identities easily.

In fact, addressing linguistic and cultural imperialism in language learning can nurture the development of critical consciousness and literacy of foreign language learners and can lead to more freedom to form a dialogical realm with native speakers based on equal stances. Through such promoted stances, they can critically analyze self-other relationships and discourses and work towards the pursuit of social justice by challenging their marginalized status and the underlying causes of oppression and inequality and hence, they can learn languages to preserve the linguistic and cultural heritages and ultimately propel them towards the path of liberation and justice. This may happen by addressing the tenets of social justice and language in future studies where the ideas of native speakerism, elitisms, and the so called prestigious inner circle languages are elaborated in future studies in a way that such ideas are no longer viewed as advantageous, unless they lead to the development of knowledge, competence, and skills among the other groups.

Future studies in the field can address language and cultural imperialism originating from more powerful local and national languages within the nation in order to see how less dominant languages are impacted and shifted due to the power relations exerted by sibling and some related languages. This can be done to improve language awareness of the people to take more care of their language and cultural identities and utilize foreign languages in more rational and non-threatening ways. These actions can be done by policy-makers and language decision makers to preserve the legal aspects of the language identity to improve their autonomy to utilize their local and national languages and save the rights of minority groups as well as nations’ and state’s roles. Moreover, through addressing power dynamics and respecting ethical implications of these reviewed studies, local and minor languages can be given more privilege to be transferred to the next generations without being threatened or endangered and the impact of language and cultural imperialism on indigenous and endangered languages can be efficiently addressed.

## References

[ref1] Ahmed I. A. Rajkhowa B. Nath A. K. (2023). Language and linguistics. Indian J. Lang. Linguist 4, 6–17. doi: 10.54392/ijll2322

[ref2] Almas N. Mazhar S. (2024). The assimilation of the native Indians by the colonizers in the United States with special reference to their languages. Remittances Rev. 9, 1869–1896. doi: 10.5325/remittancesreview.9.1.1869

[ref3] Alonso R. A. (2016). L1 influence on second language acquisition and teaching. New Trends 2, 136–149. doi: 10.18844/prosoc.v2i9.1094

[ref4] Amir Abadi S. Razmjoo S. A. (2022). A qualitative study of socio-cultural and linguistic factors affecting Iranian EFL Learners' language proficiency. J. Appl. Linguist. Stud. 1, 33–47.

[ref5] Arab H. Rastgou A. (2022). A critical analysis of Prospect 3 based on nation and Macalister's four-Strand framework. J. Appl. Linguist. Stud. 1, 58–69.

[ref6] Astanina A. Kuznetsov I. (2020). “Linguistic imperialism in EFL teaching: new role of a teacher in educating generation Z” in International scientific conference on philosophy of education, law and science in the era of globalization (PELSEG 2020) (Dordrecht, Netherlands: Atlantis Press), 16–20.

[ref7] Aydinli E. Aydinli J. (2024). Exposing linguistic imperialism: why global IR has to be multilingual. Rev. Int. Stud. 50, 943–964. doi: 10.1017/S0260210523000700, PMID: 39697825

[ref8] Ayixiren J. Zhao R. (2024). Common language development in multilingual contexts: a study of Russian language policy in the early years of the Soviet Union. J. Psycholinguist. Res. 53:2. doi: 10.1007/s10936-024-10053-0, PMID: 38240905

[ref9] Ball M. C. Bhattacharya J. Zhao H. Akpé H. Brogno S. Jasińska K. K. (2024). Effective bilingual education in francophone West Africa: constraints and possibilities. Int. J. Biling. Educ. Biling. 27, 821–835. doi: 10.1080/13670050.2023.2290482, PMID: 39723700

[ref11] Bos W. (1989). “Reliabilität und validität in der inhalts analyse [reliability and validity in content analysis]” in Angewandte inhaltsanalyse in empirischer pädagogik und psychologie [applied content analysis in empirical education and psychology]. eds. Bos W. Tarnai C. (Münster, NY: Waxmann), 211–228.

[ref12] Cheng L. S. Burgess D. Vernooij N. Solís-Barroso C. McDermott A. Namboodiripad S. (2021). The problematic concept of native speaker in psycholinguistics: replacing vague and harmful terminology with inclusive and accurate measures. Front. Psychol. 12:715843. doi: 10.3389/fpsyg.2021.715843, PMID: 34659029 PMC8517917

[ref13] Diaubalick T. Eibensteiner L. Salaberry M. R. (2023). Influence of L1/L2 linguistic knowledge on the acquisition of L3 Spanish past tense morphology among L1 German speakers. Int. J. Multiling. 20, 329–346. doi: 10.1080/14790718.2020.1841204

[ref14] Du Bois W. E. B. Césaire A. Fanon F. Said E. Wa Thiongo N. (2024). “Imperialism, colonialism and the racialised other” in Roads to decolonisation (Oxfordshire, UK: Routledge), 9–35.

[ref15] Edwards J. Edwards J. (2011). Language and imperialism. Challenges Soc. Life Lang., 161–180. London: Palgrave Macmillan. doi: 10.1057/9780230302204_8

[ref16] El-Haddad R. A. (2022). Comparative study of the changes of the language policy from colonised India to independent India. The European Conference on Education 2022: Official Conference Proceedings. 447–460. Available at: papers.iafor.org (Accessed December 4, 2024).

[ref17] Fauzan M. (2023). Imperialism ideology represented in English language teaching textbooks: a literature review. Jurnal Ilmiah Widya Borneo 6, 1–8. doi: 10.56266/widyaborneo.v6i1.142

[ref18] Foner N. (2024). Immigration and the transformation of American society: politics, the economy, and popular culture. J. Ethn. Migr. Stud. 50, 114–131. doi: 10.1080/1369183X.2023.2236906

[ref19] Golonka E. M. Bowles A. R. Frank V. M. Richardson D. L. Freynik S. (2014). Technologies for foreign language learning: a review of technology types and their effectiveness. Comput. Assist. Lang. Learn. 27, 70–105. doi: 10.1080/09588221.2012.700315

[ref20] Guo J. (2020). Study of influence of negative transfer of L1 thinking on second language acquisition. Learn. Educ. 9, 37–40. doi: 10.18282/l-e.v9i2.1393

[ref21] Huang H. T. Chan H. Y. (2024). Heritage identity and indigenous language learning motivation: a case of indigenous Taiwanese high school students. Mod. Lang. J. 108, 127–146. doi: 10.1111/modl.12894, PMID: 39721605

[ref22] Hussain S. S. (2023). The influence of L1 in English language acquisition: a case study of ESL learners at King Saud University. Rupkatha J. Interdisciplin. Stud. Human. 15, 1–24. doi: 10.21659/rupkatha.v15n2.05

[ref7004] Işık A. (2008). Yabancı dil eğitimimizdeki yanlışlar nereden kaynaklanıyor?. Journal of Language and Linguistic Studies, 4, 15–26.

[ref23] Izadpanah J. Sadighi F. Akbarpour L. (2023). The impact of gender and explicit written feedback on learners’ grammar performance. J. Appl. Linguist. Stud. 2, 92–100.

[ref24] Kalyaniwala C. Ciekanski M. (2021). Autonomy calling: a systematic review of 22 years of publications in learner autonomy and call. Lang. Learn. Technol. 25.

[ref25] Kamusella T. Ndhlovu F. (eds.) (2018). Introduction: linguistic and cultural imperialism, alas. Soc. Polit. History South. Afr. Lang., 1–11. doi: 10.1057/978-1-137-01593-8_1, PMID: 37361416

[ref7003] Kelly D. (2023). Imperialism in the academy? Challenges for academic journals. The Economic and Labour Relations Review, 34, 377–382. doi: 10.1017/elr.2023.40

[ref26] Khodadady E. Shayesteh S. (2016). Cultural and linguistic imperialism and the EIL movement: evidence from a textbook analysis. Issues Educ. Res. 26, 604–622.

[ref27] Lai M. L. (2021). English linguistic neo-imperialism–a case of Hong Kong. J. Multiling. Multicult. Dev. 42, 398–412. doi: 10.1080/01434632.2019.1702667

[ref28] Liu Y. Guo S. Gao X. (2024). Coping with National Language Policy Shift: voices of Chinese immigrant parents in an Irish County town. Br. J. Educ. Stud. 72, 457–481. doi: 10.1080/00071005.2024.2309604, PMID: 39723700

[ref29] Macaro E. Handley Z. Walter C. (2012). A systematic review of CALL in English as a second language: focus on primary and secondary education. Lang. Teach. 45, 1–43. doi: 10.1017/S0261444811000395

[ref30] Mannathukkaren N. (2023). ‘Enjoying life’: consumption, changing meanings, and social differentiation in Kerala, India. Modern Asian Studies 57, 505–554. doi: 10.1017/S0026749X22000257

[ref31] Mathew L. (2022). English linguistic imperialism from below: Moral aspiration and social mobility, vol. 28. Bristol, UK: Channel View Publications.

[ref32] McCain S. McCain S. (2018). Cultural imperialism, linguistic particularism, and local officials. Lang Question Napoleon, In T. Kamusella and F. Ndhlovu (Eds.). 65–115. doi: 10.1007/978-3-319-54936-1_3

[ref33] Mizza D. (2014). The first language (L1) or mother tongue model vs. the second language (L2) model of literacy instruction. J. Educ. Hum. Dev. 3, 101–109. doi: 10.15640/jehd.v3n3a8

[ref34] Moher D. Liberati A. Tetzlaff J. Altman D. G. The PRISMA Group (2009). Preferred reporting items for systematic reviews and meta-analyses: the PRISMA statement. PLoS Med. 151, 264–269. doi: 10.7326/0003-4819-151-4-200908180-00135, PMID: 21603045 PMC3090117

[ref35] Montrul S. (2022). “Heritage language speakers inform the critical period hypothesis for first and second language acquisition” in Generative SLA in the age of minimalism. Features, interfaces, and beyond. Selected proceedings of the 15th generative approaches to second language acquisition conference (Amsterdam, the Netherlands: John Benjamins Publishing Company), 265–286.

[ref36] Murakami A. Alexopoulou T. (2016). L1 influence on the acquisition order of English grammatical morphemes: a learner corpus study. Stud. Second. Lang. Acquis. 38, 365–401. doi: 10.1017/S0272263115000352

[ref37] Namboodiripad S. Henner J. (2022). Rejecting competence–essentialist constructs reproduce ableism and white supremacy in linguistic theory: a commentary on “undoing competence: coloniality, homogeneity, and the overrepresentation of whiteness in applied linguistics”. Lang. Learn. 73, 321–324. doi: 10.1111/lang.12534

[ref38] Nitisakunwut P. Nutayangkul T. Liang-Itsara A. (2023). The sociocultural perspective on the use of L1 as a supporting tool for EFL learning. Engl. Lang. Teach. 16, 74–81. doi: 10.5539/elt.v16n2p74

[ref39] Onanuga P. A. (2024). Asserting identity in stifling spaces: multisemioticity in Nigerian queer-positive Instagram. Soc. Dyn., 1–25. doi: 10.1080/02533952.2024.2320579

[ref40] Oviedo D. C. (2017). Theoretical paradigms on L1 acquisition, Revista Vinculando. 15. Available at: https://vinculando.org/en/theoretical-paradigms-on-l1-acquisition.html

[ref41] Philips M. (1987). Potential paradigms and possible problems for CALL. System 15, 275–287. doi: 10.1016/0346-251X(87)90002-9

[ref42] Phillipson R. (1992). Linguistic imperialism. Oxford: Oxford University Press.

[ref7001] Phillipson R. (2008). The Linguistic Imperialism of Neoliberal Empire. Critical Inquiry in Language Studies, 5, 1–43. doi: 10.1080/15427580701696886

[ref43] Phillipson R. (2009). Linguistic imperialism continued. London: Routledge.

[ref44] Phillipson R. (2012). “Imperialism and colonialism” in The Cambridge handbook of language policy. ed. Spolsky B. (Cambridge: Cambridge University Press), 203–235.

[ref45] Phillipson R. (2014). Reflections by Robert Phillipson on English in post-revolutionary Iran: from Indiginazation to internationalization. Int. J. Soc. Lang. Cult. 2, 131–137.

[ref46] Phillipson R. Kabel A. (2024). “Linguistic imperialism in English-medium higher education” in The Routledge handbook of English-medium instruction in higher education (London: Routledge), 63–77. doi: 10.4324/9781003011644-6

[ref47] Pujasari R. S. Hikmatullah N. (2023). A new paradigm on language imperialism: student-teachers voice on English language learning. Script J. 8, 73–82. doi: 10.24903/sj.v8i01.1092

[ref48] Rose H. Conama J. B. (2018). Linguistic imperialism: still a valid construct in relation to language policy for Irish sign language. Lang. Policy 17, 385–404. doi: 10.1007/s10993-017-9446-2

[ref49] Saba T. Siddiqui B. (2023). Portrayal of the ‘other’: tracing the linguistic imperialism in English textbooks of matric and intermediate. J. Develop. Soc. Sci. 4, 95–109. doi: 10.47205/jdss.2023(4-II)10, PMID: 39685847

[ref50] Sah P. K. Fang F. (2024). Decolonizing English-medium instruction in the global south. TESOL Q. doi: 10.1002/tesq.3307

[ref51] Schlechtweg M. Peters J. Frank M. (2023). L1 variation and L2 acquisition: L1 German/eː/−/ɛː/overlap and its effect on the acquisition of L2 English/ɛ/−/æ. Front. Psychol. 14:1133859. doi: 10.3389/fpsyg.2023.1133859, PMID: 37448717 PMC10336208

[ref52] Shokri H. (2022). Comparative critical gender analysis in Iranian news papers between Persian and English art and culture sections. J. Appl. Linguist. Stud. 1, 47–56.

[ref53] Singh M Gaur R. (2020). Politics of language and countering colonialism: A study of Amitav Ghosh’s the shadow lines. In Linguistic Foundations of Identity. 68–78. Routledge. doi: 10.4324/9781003106807-4

[ref54] Soto-Molina J. E. Méndez P. (2020). Linguistic colonialism in the English language textbooks of multinational publishing houses. HOW 27, 11–28. doi: 10.19183/how.27.1.521

[ref55] Spolsky B. (2019). A modified and enriched theory of language policy (and management). Lang. Policy 18, 323–338. doi: 10.1007/s10993-018-9489-z

[ref56] Sugiharto S. (2024). Translanguaging as a counter-narrative in EFL practice. EDULANGUE 6, 138–149. doi: 10.20414/edulangue.v6i2.9138

[ref57] Tajfel H. Turner J. C. (2004). “The social identity theory of intergroup behavior” in Political psychology (London, England: Psychology Press), 276–293.

[ref58] Thao M. T. P. (2020). Role of L1 in L2 acquisition according to contrastive analysis hypothesis and error analysis. J. Educ. Pract. 11, 102–107. doi: 10.53935/2641-533x.v3i2.142

[ref59] Udoh I. Emmanuel I. (2020). Nigerian languages and identity crises. Lang. Semiotic Stud. 6, 96–111. doi: 10.1515/lass-2020-060305

[ref7002] Van Dongera R. VanderMeer C. Sterk R. (2017). Research for CULT Committee-Minority languages and education: Best practices and pitfalls. European Parliament, Policy Department for Structural and Cohesion Policies, Brussels.

[ref60] Wang Z. (2017). “A sociocultural perspective on the role of L1 in L2 learning: a comparative analysis of two L2 studies” in Proceedings of the 2017 international conference on education and E-learning, 77–80. (New York, USA).

[ref61] White M. J. Southwood F. Huddlestone K. (2023). Children’s acquisition of negation in L1 Afrikaans. First Lang. 43, 22–57. doi: 10.1177/01427237221112064

[ref62] Yamada E. (2023). “The views on linguistic imperialism in multicultural classroom” in The IAFOR international conference on Arts & Humanities–Hawaii 2023 official conference proceedings. The International Academic Forum (IAFOR). A graduate school of Osaka University, Japan.

[ref63] Yazovskaya O. V. Gudova I. V. (2020). Notions of empire and cultural imperialism in the postcolonial discourse. KnE Soc. Sci., 76–82. doi: 10.18502/kss.v4i13.7699, PMID: 39712361

[ref64] Yook C. (2012). L1 influence on ESL Learners' Acquisition of English ditransitive constructions. English Teach. 67, 27–50. doi: 10.15858/engtea.67.2.201207.27

[ref65] Zeng J. Ponce A. R. Li Y. (2023). English linguistic neo-imperialism in the era of globalization: a conceptual viewpoint. Front. Psychol. 14:1149471. doi: 10.3389/fpsyg.2023.1149471, PMID: 36968741 PMC10032042

[ref66] Zeng J. Yang J. (2024). English language hegemony: retrospect and prospect. Human. Soc. Sci. Commun. 11, 1–9. doi: 10.1057/s41599-024-02821-z, PMID: 39310270

[ref67] Zou D. Luo S. Xie H. Hwang G. (2020). A systematic review of research on flipped language classrooms: theoretical foundations, learning activities, tools, research topics and findings. Comput. Assist. Lang. Learn. 35, 1811–1837. doi: 10.1080/09588221.2020.1839502, PMID: 39723700

[ref68] Ztf P. B. (2017). Language and cultural imperialism: Indonesian case. Int. J. Lang. Res. Educ. Stud. 1, 165–172. doi: 10.30575/2017081212

