# Supplementary material for: The impact of linguistic vs. cultural imperialism on language learning
Source: Front Psychol. 2025 Jan 29;15:1438849. doi: 10.3389/fpsyg.2024.1438849 (PMC11814222; doi:10.3389/fpsyg.2024.1438849)
Supplement: Supplementary file 1 [file Data_Sheet_1.docx]

**Appendix A: Summary of the Main Characteristics of Articles**

| Key Findings | Identified significant influence of cultural practices on language preservation and shift. Highlighted the role of community engagement in language revitalization efforts | English dominance creates a dependent relationship between an English-speaking core and a non-English periphery, impeding original contributions from the periphery. | Bilingual education shows potential for improving literacy and cognitive skills, but faces constraints such as lack of resources, teacher training, and policy support. | High L2 proficiency helps learners match native speakers’ choices in prototypical contexts. However, in non-prototypical contexts, L2 knowledge is less beneficial due to higher processing costs. | Highlights how imperialism and colonialism have shaped modern racial identities and subjectivities, emphasizing the need for decolonization and the recognition of diverse epistemologies |
| --- | --- | --- | --- | --- | --- |
| Instruments | Interviews, participant observations, and document analysis | Journal analysis, citation analysis, and ranking assessment | Surveys, interviews, classroom observations, and standardized test scores | Written completion tasks to assess the use of Spanish past tense morphology in various contexts. | Conceptual frameworks such as Du Bois’ “double consciousness” |
| Design | Qualitative research using case studies and ethnographic methods | Analyzes 135 journals from 39 countries in the linguistic periphery to assess English dominance. | Mixed-methods approach, including interviews and quantitative analysis | Analyzes data from 73 German-speaking learners, based on their proficiency level | Theoretical analysis drawing on historical events |
| Context | Examines the impact of socio-cultural factors on language use and development in multilingual communities | Examines the dominance of English in International Relations and its impact on global IR knowledge production | Investigates the effectiveness of bilingual education in Francophone West Africa, focusing on the challenges and opportunities | Investigates how L1 (German) and L2 (another Romance language) linguistic knowledge influences the acquisition of L3 (Spanish) past tense | Explores the relationship between imperialism, colonialism, and the construction of the racialised subject |
| Publication source | Indian J. Lang. Linguist | Review of International Studies | International Journal of Bilingual Education and Bilingualism | International Journal of Multilingualism | Roads to Decolonisation, Routledge |
| Authors | Ahmed, Rajkhowa, & Nath (2023) | Aydinli & Aydinli (2024) | Ball, Bhattacharya, Zhao, Akpé, Brogno, & Jasińska (2024). | Diaubalick, Eibensteiner, & Salaberry (2023) | Du Bois, Césaire, Fanon, Said, & wa Thiong'o (2024). |

| Key Findings | Identified the persistence of English as a dominant language and the challenges in promoting local vernaculars. Provided recommendations for enhancing the value of local languages in education | Identified the prevalence of Western cultural dominance and the marginalization of local cultures in textbooks. Suggested the need for more inclusive and culturally sensitive teaching materials | Found that L1 facilitates learning and cannot be entirely eliminated in ESL classrooms. Identified common language errors and the importance of understanding cognates | Highlighted the marginalization of indigenous languages and the dominance of colonial languages. Emphasized the need for policies that support linguistic diversity and cultural preservation | English schooling is driven by social mobility needs rather than leading to it. The study highlights the moral and social dimensions of aspirations for English education, showing how these aspirations are embedded in broader socio-political and economic contexts |
| --- | --- | --- | --- | --- | --- |
| Instruments | Historical documents, policy analysis, and educational records | Content analysis and critical discourse analysis of textbooks | Open-ended questionnaires and Chi-square tests | Historical and sociolinguistic analysis, policy review | Interviews, home visits, classroom observations, and analysis of textbooks and policy |
| Design | Comparative analysis of historical and contemporary language policies in India | Literature review of existing studies on the topic | Case study involving survey data from 54 ESL learners | Interdisciplinary analysis of social and political histories of Southern African languages | Ethnographic study involving two schools |
| Context | Examines the impact of British imperialism on India’s language policy and its evolution post-independence | Investigates how imperialist ideologies are embedded in English language teaching textbooks | Examines the role of the first language (L1) in acquiring (ESL) among students at King Saud University | Explores the impact of linguistic and cultural imperialism on the languages of Southern Africa | Examines the role of English in social mobility and moral aspiration in Kerala, India |
| Publication source | Changes of the Language Policy | Jurnal Ilmiah Widya Borneo. | Rupkatha Journal on Interdisciplinary Studies in Humanities | The Social and Political History of Southern Africa's Languages | Channel View Publications |
| Authors | El-Haddad, (2022) | Fauzan, (2023) | Hussain, S. S. (2023). | Kamusella & Ndhlovu, (2018) | Mathew, (2022). |

| Key Findings | English maintains hegemonic power, leading to perceptions of linguistic exclusion and neo-imperialism | Increasing integration into global markets has transformed consumption patterns. | Reveals the tension between cultural imperialism and linguistic particularism, highlighting local officials’ roles as cultural intermediaries | Argues for replacing the term “native speaker” with more inclusive and accurate measures to avoid perpetuating normative assumptions and marginalizing certain populations | Argues that essentialist constructs like competence perpetuate ableism and white supremacy, advocating for more inclusive and equitable linguistic practices |
| --- | --- | --- | --- | --- | --- |
| Instruments | Critical analysis of emails and documents related to the language conflict | Interviews, surveys, and analysis of consumption patterns | Analysis of linguistic surveys and reports conducted by the French Bureau de la statistique | Analysis of existing literature and terminology used in psycholinguistic research | Review of linguistic theories and constructs, particularly those related to competence |
| Design | Qualitative case study focusing on a specific language conflict at a university in Hong Kong | Fieldwork-based study supplemented with quantitative data | Historical analysis of correspondence and documents | Policy and practice review within the field of psycholinguistics | Theoretical commentary based on existing literature and critical analysis |
| Context | Examines the dominance of English in Hong Kong post-colonial era, highlighting linguistic conflicts | Examines the changing meanings of consumption and its role in social differentiation in Kerala, India | Examines the Napoleonic state’s efforts to standardize language and the role of local officials in this process | Critiques the use of the term “native speaker” in psycholinguistics, highlighting its vagueness and harmful implications | Commentary on the critique of competence in applied linguistics, focusing on its colonial and exclusionary implications |
| Publication source | Journal of Multilingual and Multicultural Development | Modern Asian Studies | The Language Question | Frontiers in Psychology | Language Learning |
| Authors | Lai, (2021) | Mannathukkaren, (2023) | McCain & McCain (2018) | Cheng et al. (2021) | Namboodiripad & Henner (2022) |

| Key Findings | Highlights the benefits of using L1 to support cognitive tasks, improve comprehension, and facilitate discussion and reasoning in EFL learning | Nigerian queer users employ a variety of semiotic resources to navigate and resist heteronormative pressures | EMI can lead to linguistic imperialism, marginalizing local languages and cultures, and reinforcing global inequalities | Student-teachers view teaching English as a form of empowerment, not imperialism, and believe it helps students navigate a globalized world while promoting local cultures | Highlights discrimination and inequality faced by ISL users, fitting the linguistic imperialism paradigm, including linguicism, audism, and denial of linguistic rights |
| --- | --- | --- | --- | --- | --- |
| Instruments | Analysis of existing literature and case studies on L1 use in EFL settings | Analysis of visual, textual, and multimodal elements in Instagram posts | Review of existing literature and policy documents on EMI | Interviews and qualitative analysis of responses | Review of policy documents and literature on ISL and linguistic imperialism |
| Design | Review of socioculturally informed research on L1 use in EFL classrooms | Qualitative study using multisemiotic analysis of Instagram posts | Theoretical analysis of English-medium instruction (EMI) policies and practices | Descriptive qualitative study involving interviews with six student-teachers | Theoretical analysis within a conceptual framework of linguistic imperialism |
| Context | Investigates the role of the first language (L1) in supporting EFL learning from a sociocultural perspective | Examines how Nigerian queer individuals assert their identities on Instagram | Investigates the dominance of English in higher education and its impact on other languages | Explores student-teachers’ perspectives on English language learning in Indonesia imperialism | Analyzes the application of linguistic imperialism to Irish Sign Language (ISL) policy |
| Publication source | English Language Teaching | Social Dynamics | The Routledge Handbook of English-Medium Instruction in Higher Education | Journal of Linguistics and English Teaching | Language policy |
| Authors | Nitisakunwut, Nutayangkul, & Liang-Itsara (2023) | Onanuga (2024) | Phillipson, & Kabel (2024) | Pujasari, & Hikmatullah (2023) | Rose, & Conama (2018). |

| Key Findings | English textbooks promote Western ideologies and cultural norms, reinforcing the dominance of English and marginalizing local languages and cultures | EMI often perpetuates colonial ideologies and unequal power dynamics. The study calls for a critical examination of these dynamics and suggests that EMI programs may inadvertently benefit privileged groups and nation-states | Maintaining the vowel contrast in L1 German leads to more native-like identification of /æ/ in L2 English. Sociolinguistic variation in L1 can significantly impact L2 acquisition | The study highlights how Ghosh uses language to challenge colonial narratives and promote a decolonial perspective. | Textbooks often contain high levels of cultural alienation and superficial cultural content, reinforcing the dominant culture of English |
| --- | --- | --- | --- | --- | --- |
| Instruments | Analysis of linguistic devices and cultural content in the textbooks | Literature review, policy analysis, and case studies from different countries in the Global South | Sound files for identification and discrimination tasks, logistic regression models, and linear mixed effects models to analyze the data | Textual analysis, examining narrative techniques, character dialogues, and thematic elements | Textbook content analysis, focusing on cultural themes and their representation |
| Design | Thematic analysis of English textbooks using Phillipson’s Linguistic Imperialism framework | Qualitative analysis using a decolonial lens to critique EMI policies and practices | Conducted identification and discrimination experiments with German learners of English to assess their ability | Literary analysis focusing on the themes of language, colonialism, and identity | Documentary analysis of six textbooks, comparing their cultural theories components |
| Context | Investigates how English textbooks for Matric and Intermediate in Pakistan portray linguistic imperialism and its impact on cultural ideologies | Examines the adoption of English-medium instruction (EMI) in the Global South, highlighting the colonial legacy and its impacts on education systems | Investigates how variations in the (L1) German vowel sounds affect the acquisition of second language (L2) English vowel | Analyzes the politics of language and its role in countering colonialism through the lens of Amitav Ghosh’s novel | Examines linguistic colonialism and cultural alienation in English language textbooks |
| Publication source | Journal of Development and Social Sciences | TESOL Quarterly | Frontiers in Psychology | Linguistic Foundations of Identity | HOW |
| Authors | Saba & Siddiqui, (2023) | Sah & Fang, (2024) | Schlechtweg Peters & Frank (2023) | Singh & Gaur, (2020) | Soto-Molina & Méndez, (2020) |

| Key Findings | Translanguaging promotes linguistic inclusivity and empowers students by validating their linguistic repertoires. It challenges the dominance of English-only policies and supports more equitable language practices in EFL classrooms | L1 influences L2 acquisition through transfer, interference, and error patterns | Identifies various forms of modern cultural imperialism and their impacts on global interaction | Highlights how English maintains its dominance in communication, business, academia, and education, and discusses implications for local languages | English’s dominance impacts cultural, educational, and technological domains globally, with implications for language policies in non-native English-speaking countries |
| --- | --- | --- | --- | --- | --- |
| Instruments | Classroom observations, teacher interviews, and analysis of student work | Literature review and theoretical analysis | Literature review of key postcolonial theorists and concepts | Literature review and theoretical framework analysis | Literature review and critical analysis of linguistic hegemony theories |
| Design | Qualitative study analyzing classroom practices and teacher interviews to understand the implementation and impact of translanguaging in EFL settings | Comparative analysis of CAH and EA | Theoretical analysis of postcolonial theories and modern imperialism | Conceptual analysis of linguistic neo-imperialism | Socio-linguistic approach combined with historical and interdisciplinary analysis |
| Context | Explores the use of translanguaging as a counter-narrative in (EFL) practice, challenging traditional monolingual approaches | Examines the role of the first language (L1) in second language (L2) acquisition | Explores theoretical approaches to empire and cultural imperialism in postcolonial discourse | Examines the spread and dominance of English in the era of globalization | Explores the historical development and future prospects of English language hegemony |
| Publication source | Edulangue | Journal of Education and Practice | KnE Social Sciences | Frontiers in Psychology | Humanities and Social Sciences Communications |
| Authors | Sugiharto (2024) | Thao (2020) | Yazovskaya & Gudova, (2020) | Zeng, Ponce, & (2023) | Zeng & Yang, (2024) |
